# Effect of bonding characteristics of major constituents of mineral filler-based glass fiber reinforced with epoxy composites

**DOI:** 10.1038/s41598-025-04354-0

**Published:** 2025-07-01

**Authors:** K. S. Lokesh, C. G. Ramachandra, Thandra Pavan Kumar, Praveen Kumar Kanti, Prabhu Paramasivam, Abinet Gosaye Ayanie

**Affiliations:** 1 Department of Aeronautical Engineering, Srinivas Institute of Technology, Mangalore, Karnataka 574143 India; 2 Department of Mechanical Engineering, Presidency University, Bengaluru, India; 3 University Center for Research & Development (UCRD), Chandigarh University, Mohali, Punjab India; 4 Department of Research and Innovation, Saveetha School of Engineering, SIMATS, Chennai, Tamil Nadu 602105 India; 5 Department of Mechanical Engineering, Adama Science and Technology University, Adama, 2552 Ethiopia

**Keywords:** Computational study, Epoxy, Bonding, NMR study, Filler composites, Engineering, Mechanical engineering

## Abstract

In the present study, attempt has been made in understanding the bonding behaviour of mineral filler when it is introduced with epoxy matrix structured with e-glass fibre at molecular level. Firstly, filler content in GFRP composite was analysed through Fourier Transform infrared spectroscopy (FTIR). Here, Silicon dioxide has been chosen as a representative for E-glass fibre as Silicon dioxide holds major part in the composition of an E-glass fibre. DFT simulation techniques has been employed to study the reaction in between them. In order to increase the binding capability, wollastonite has been introduced into the system and many possible configurations were modelled for study. Out of all the models, the model with the highest dipole moment and stability has been considered. Spectral studies such as NMR, VCD and IR studies has been done to witness the oxygen atoms in the glass fibre acted as the connecting bridge in between the silicon atoms of the glass fibre and the carbon atoms of the epoxy resin. But these alone were not enough to obtain a stable structure that was described above. The calcium atoms in the wollastonite acted as better electron bridges and support for the complex. This work majorly focusses on the interactions between epoxy resin(ly556) and SiO_2_ molecule and the filler material wollastonite (CaSiO_3_).

##  Introduction

Fibre reinforced plastics remain an interesting choice of materials and often pose challenges when explored them with different combination of fibres and fillers, resulting in variations in their properties^1–5^. Experimental studies on composite composition using characterization techniques are a significant method to interpret the features of polymer-based materials^6–9^. A study of the behavioural existence of core elements necessitates the use of modern tools to unveil their properties at the molecular level^10–13^. One such study involves computational analysis for developed composites, typically used to calculate atomic-scale interactions of the molecules under study^14–17^. Ground state optimizations were carried out using the Quantum Espresso ab initio simulation package for epoxy resin (LY556), which is based on a bisphenol-A epoxy molecule. The simulation also included silicon dioxide, representing the atomic structure of E-glass fibre, and calcium silicate. These calculations employed the Perdew-Burke Ernzerh (PBE) functional with ultra-soft pseudopotential^18,19^. The cut-off energy for the plane-wave basis set was adjusted to 25 Ry. During geometry optimization, all the atoms in the structure are allowed to relax until no residual forces remained between them. Gamma functions were employed for K-point mesh grid, and the interaction energies were calculated accordingly^20^. To identify the nature of interactions between the epoxy molecule and both silica and wollastonite, the structures were modelled by placing silica and wollastonite on different electrophilic binding sites of the epoxy. Among all configurations, the most stable structure- with the best dipole moment was selected^21,22^. With the help of these equilibrium geometries, different structure-based parameters and interaction energies were calculated^23^. The first phase of the calculation involved analysing the structural stability of epoxy molecule. In the second phase, the structure modified by the SiO_2_ was studied. The third phase focussed on calculating the interaction energy and structural properties of the final sample, which ws the wollastonite enhanced epoxy-silica fibre. Keeping all these aspects in mind, present study aims to investigate, at the molecular level, bonding behaviour of epoxy resin with mineral fillers (SiO_2_ and CaSiO_3_) within glass fiber reinforced polymer (GFRP) composite. Using DFT simulations and spectral analyses (NMR, VCD, IR), the study focuses on understanding how wollastonite improves interfacial bonding by facilitating stronger interactions between the epoxy matrix and the E-glass fiber, enhancing composite stability.

## Material and experimentation

**Fig. 1:**
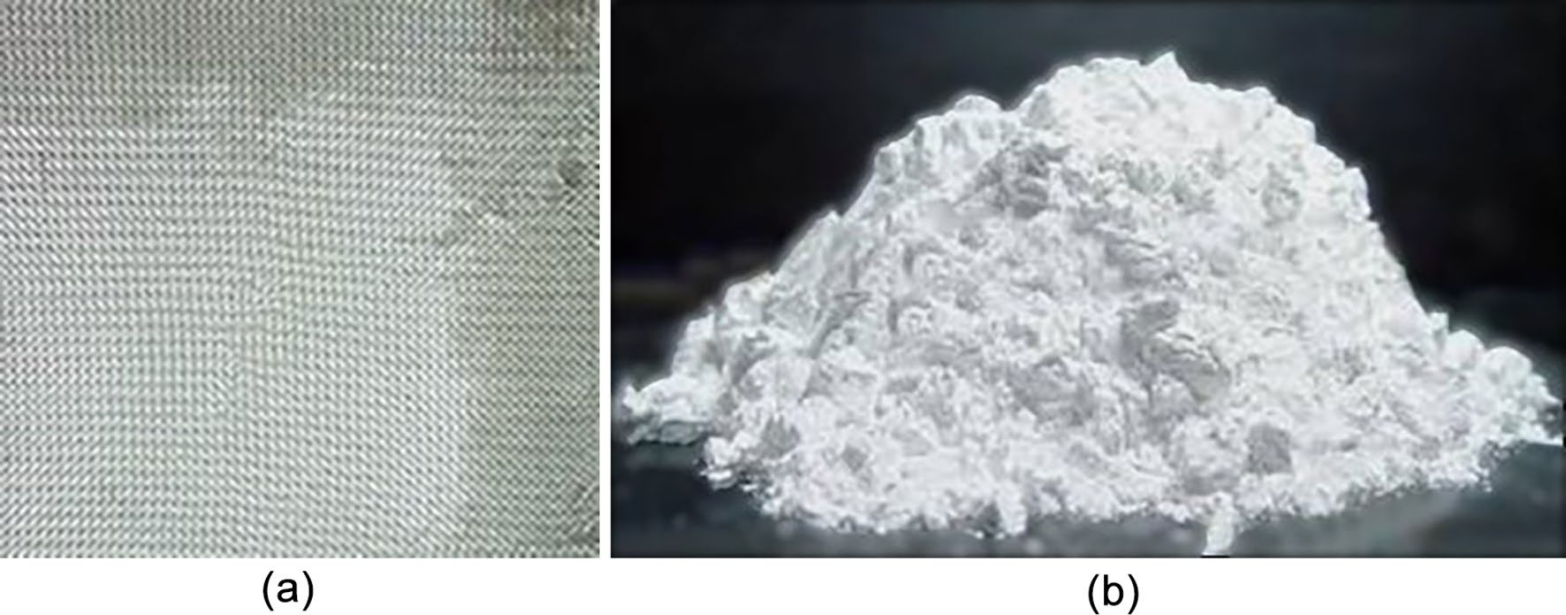
(**a**) Woven roving E-Glass fibre (**b**) Calcium-Ino silicate filler (Wollastonite).

To develop a filler-based composite, E-glass as shown in Fig. 1(a) & epoxy (Ly556) as binder both procured from Shreyas polymers Bangalore was used as major constituents. Mineral filler with grain size of 250 μm procured from AMGEEN minerals from Vadodara, Gujarat is used as a filler.

The exact composition of e-glass fibre with resin used here it is 60:40 by adding varied percentage by weight of filler by 1%,3%,5% and 7% and mixing was done by mechanical stirring for the duration of 5 min, once samples were prepared, compression was done with load capacity of 5kN, samples were cured at room temperature conditions for the post-curing duration of 48 h.

**Fig. 2:**
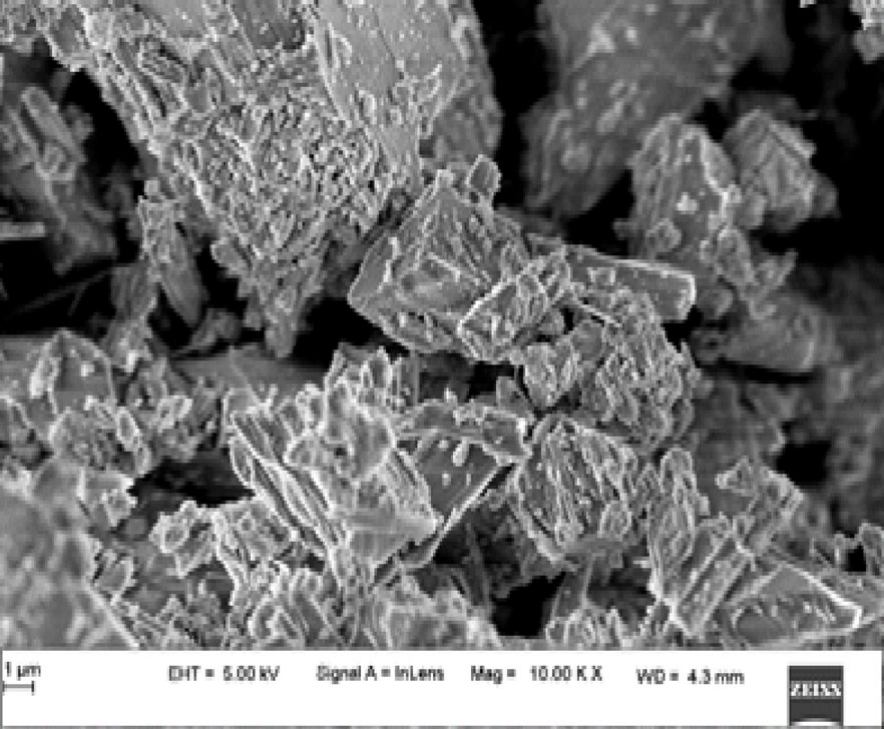
Microstructure of calcium-ino silicate.

Details of materials used is shown in Table 1. Microstructure of the Calcium-Ino silicate filler as shown in Fig. 1 (b) also termed as wollastonite is as shown in figure.2.

**Table 1:** Materials used.

Fibre	E-glass (Bi-directional Fabric)
Resin	Epoxy (LY 556)
Hardener	HY 917
Filler	Calcium-Ino silicate (wollostonite)

**Properties of Calcium-Ino silicate filler**

- Colour:- White.
- PH:- 9.9.
- Hardness:- 4–5 Mohs.
- Density:- 2.94 g/cc.
- Specific gravity:- 2.87–3.09.
- Composition48.28% CaO & 51.72% SiO_2_ .

### Sample preparation

In order to perform compositional analysis through FTIR, samples were prepared through manual layup route, and mineral filler was added with filler weight% of 1,3,5 and 7% to epoxy binder through manual mixing. Resulting composites were analysed through FTIR to compute the results further. FTIR device model used here in the present study is IRTracer-100 supplied from Shimadzu where NMR device model is JNM-ECZL series supplied from JEOL and Raman spectroscopy model used is DXR3 Raman Microscope supplied from Thermo Fisher Scientific firm.

###  Computational analysis

The current computational analysis is performed to calculate the atomic scale interactions of the molecules under study. Ground state optimizations for epoxy resin (LY556) which is based on an epoxy molecule made from bisphenol-A, silicon dioxide which is the atomic representation of the E-glass fibre and wollastonite, is carried out using Quantum Espresso ab initio simulation package, employing the Perdew-Burke Ernzerh (PBE) functional with ultra-soft pseudopotential.

The cut-off energy for the plane wave basis set was adjusted to 25 Ry, the cut-off energy in plane-wave DFT, such as the ones in Quantum Espresso, defines the kinetic energy threshold for including plane waves in the wave function expansion, which is given by h^2^/2m[k + G]^2^ where k is the wave vector and G is the reciprocal lattice vector. A higher cut-off increases accuracy but comes with a computational cost. The choice for 25 Ry is a part of the standard practice when using ultrasoft-pseudopotentials. This ensures convergence for Si, O and Ca. The typical cut-off energies for systems like this lies in between 25 and 40 Ry as seen in similar studies^24^. Geometric optimization has been done so that all the atoms in the structure are allowed to relax until there are no more residual forces left in between them, gamma functions were employed for K-point mesh grid and the interaction energies were calculated accordingly. To identify the nature of interactions of the epoxy molecule with the silica and wollastonite, the structures were modelled by placing the silica and the wollastonite molecule on different electrophilic binding sites of the epoxy molecule and out of all the possible configurations, the best structure with a better dipole moment and stability was picked out. The nominal units used here is Rydberg (Ry) where it is defined as the ionization energy of hydrogen in the Bohr model, given by -

$$\:1\:Ry\:=\:hc{R}_{\infty\:}\:\approx\:\:13.605\:eV\:=\:2.179\:\times\:\:{10}^{\:-18}\:J$$

Plane wave codes such as the Quantum Espresso use Ry as their native unit for both the plane-wave cut-off and total energies, since it directly ties to the fundamental Rydberg constant with the help of these equilibrium geometries, different structure-based parameters and interaction energies were calculated. The first phase of the calculation includes the structural stability analysis of the epoxy molecule, the second phase being the structure modified by the SiO_2_ and its respective analysis, the third phase was the calculation of the interaction energy and the structural properties of the final sample which is the wollastonite enhanced epoxy-silica fibre. Their respective parameters are compared and are presented below. Simulation based spectral studies for the final sample was carried out using Gaussian 9 software employing F-OPT simple calculation with For the purpose of optimising the ground state structure, the present is done based on the Density Functional Theory (DFT) using Becke’s three-parameter functional^20^ and the Lee-Yang-Parr functional (B3LYP) and a 6-31G (d, p) basis set.

## Results and discussion

### Chemical analysis

**Fig. 3:**
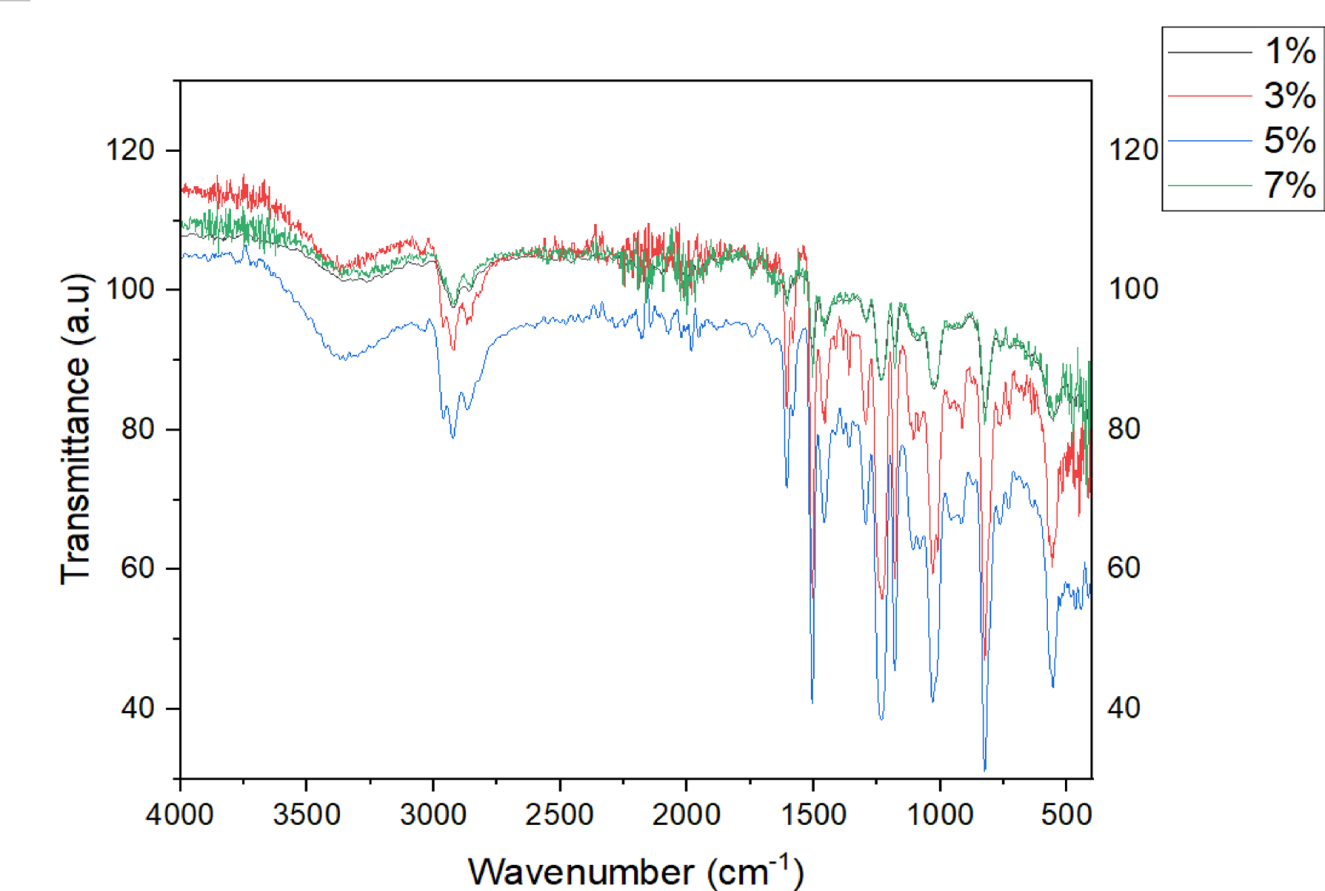
FTIR spectra for different filler composition.

FTIR spectra was carried out to all the samples with different compositions of the filler material (1%, 3%, 5%, 7%) to compare the bonding nature in between the samples and to derive a reason for the increased mechanical and structural properties in some samples and decreased activity in others. The fingerprint region was taken from 2000 cm^− 1^. and the peaks observed in the functional group region were broader in nature. Figure 3 shows sample with 1% filler shows us that the peak at 3292 cm^− 1^ indicates that there is a presence of methylene N-H bond stretch which indicates the presence of the hardener Triethylenetetramine (TETA).

**Table 2:** Summary of identified wavenumbers and their corresponding features.

Identified Wave number (cm^− 1^)	Vibration / Bond Type	Possible Features
3932	N-H stretch	Methylene groups in TETA hardener
2700–3200	O-H stretch (Hydrogen-Bonded)	Hydroxyl groups in epoxy resin
1606.70	C = C-C stretch	Aromatic ring in the epoxy resin
1504.48	C = C-C stretch	Aromatic ring in epoxy resin
1456.26	C = C-C stretch	Aromatic ring in epoxy resin
1232	Si-O-Si anti-symmetric vibration	Interaction between epoxy resin and silicon-containing components (e.g- wollastonite or E-glass fibres)
1031.92	Si-O-Si anti-symmetric vibration	Interaction between epoxy resin and silicon-containing components (e.g- wollastonite or E-glass fibres)
1024.20	Si-O-Si anti-symmetric vibration	Interaction between epoxy resin and silicon-containing components (e.g- wollastonite or E-glass fibres)
823.60	C-O-O stretch	Possible linear peroxide bridge and silicon-containing components (e.g. wollastonite or E-glass fibers
400–600	Si-O flexural vibration, Ca-O stretch	Presence of wollastonite (CaSiO_3_ ) and E-Glass fibers

**Fig. 4:**
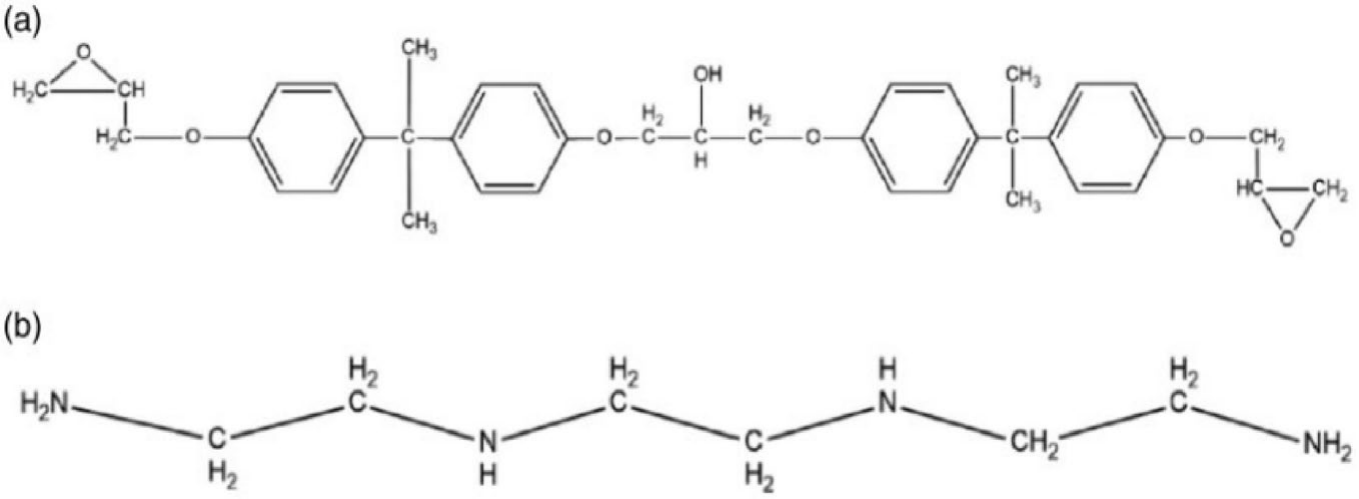
(**a**) Epoxy structure (**b**) Tri ethylene-tetramine Hardener.

The bands occurring at the region from 2700 to 3200 cm^− 1^ depicts that there is a hydrogen bonded OH stretch with a broader peak on the other hand if the peak is a narrow one, then it can be a band which is a normal polymeric OH stretch. The bands occurred at 1606.70 cm^− 1^, 1504.48 cm^− 1^ and 1456.26 cm^− 1^ indicate that there is a presence of an aromatic ring with a C = C-–C stretch that points out to the phenyl ring present in the epoxy resin sample. The bands here also point out the formation of the bond formed between the aliphatic and the aromatic compounds present in the samples. The band at 1232 cm^− 1^ indicates that there is an aromatic ether as seen in the above Table 2. Absorption band with 1024.20 cm^− 1^ and 1031.92 cm^− 1^ indicate the presence of a silicon bonding which was caused by Si-O-Si antisymmetric vibrations. These indicate that there has been an interaction between the epoxy resin and wollastonite which are in turn connected to the e-glass fibers. Bands produced in the range of 400–600 cm^− 1^ are caused by the Si-O flexural vibrations and Ca-O stretching vibrations and the presence of a halo compound, the reason being their appearances at low wave numbers. The bands occurring at the lower wave numbers are difficult to categorize as they can be organic and inorganic in nature and their formation can only be confirmed if they appear again at a bigger wave number. Linear peroxide stretch (C-O-O) can be identified at lower band level value of 823.60 cm^− 1^. The bands appearing at this level can indicate that there was a possible linear peroxide stretch which has connected epoxy resin molecule and the silicon content of the glass fiber which are bridged by the oxygen atoms. By looking with structural compositions of the binder LY556 with hardener TETA, it is possible to confirm that the bonds have been observed. The formula that denotes TETA hardener is C_6_H_18_N_4_, and that of epoxy matrix is C_18_H_21_ClO_3_. Their chemical structures depicted in figure.5.7 demonstrate existing N-H, O-H & C-N bonds that are stretched as well as bending C-H bonds. Chemical bonds demonstrated via graphical representation stands collinear with structure examined over FTIR delves in to accuracy of the analysis made. Figure 4 depicts the (a). Epoxy structure (b). TETA Hardener.

###  Molecular modelling of calcium-ion silicate filled E-glass fiber polymers

**Fig. 5:**
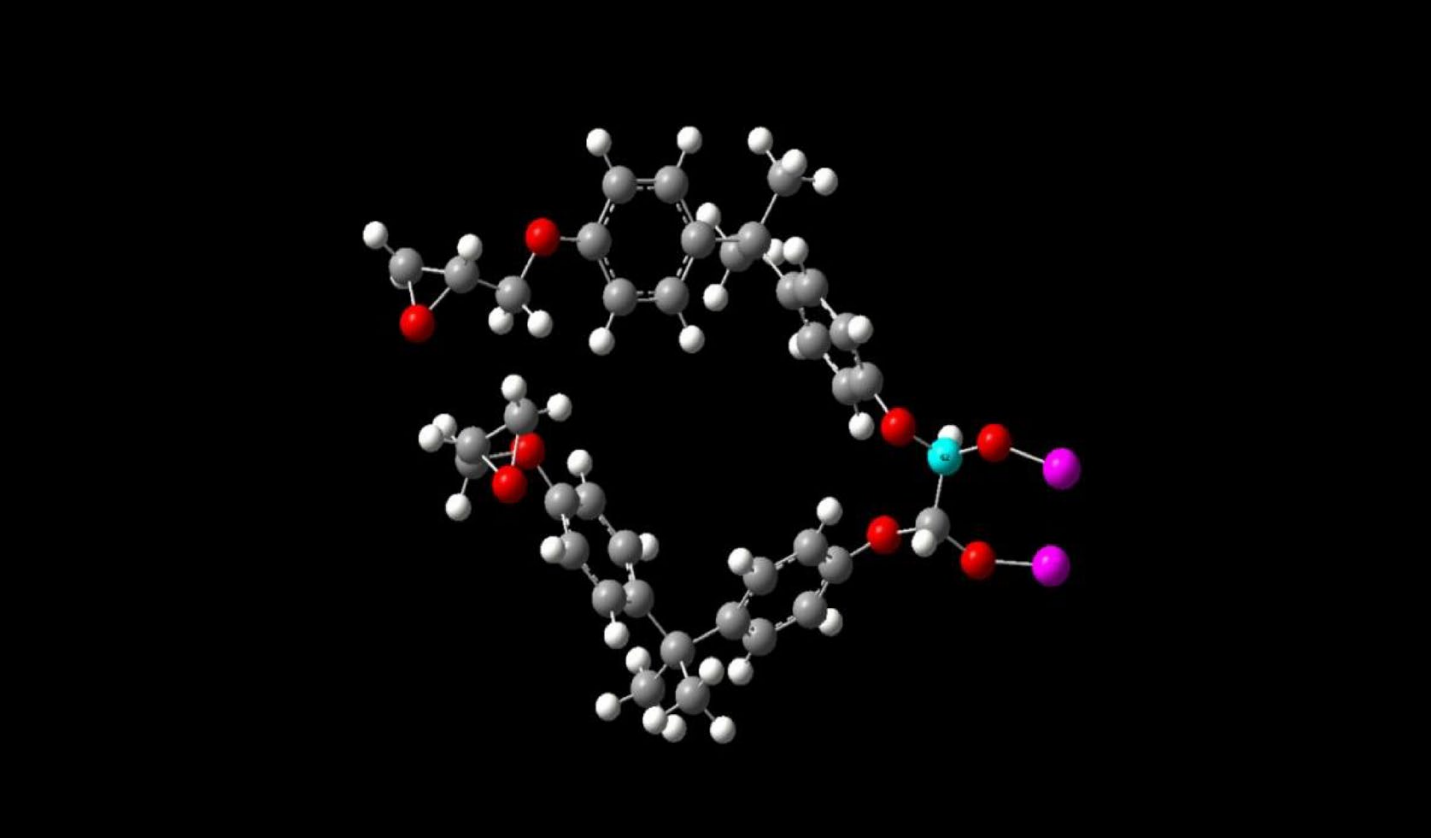
Molecular representation of Bisphenol-A, illustrating the epoxy resin LY 556 structure without the filler.

**Fig. 6:**
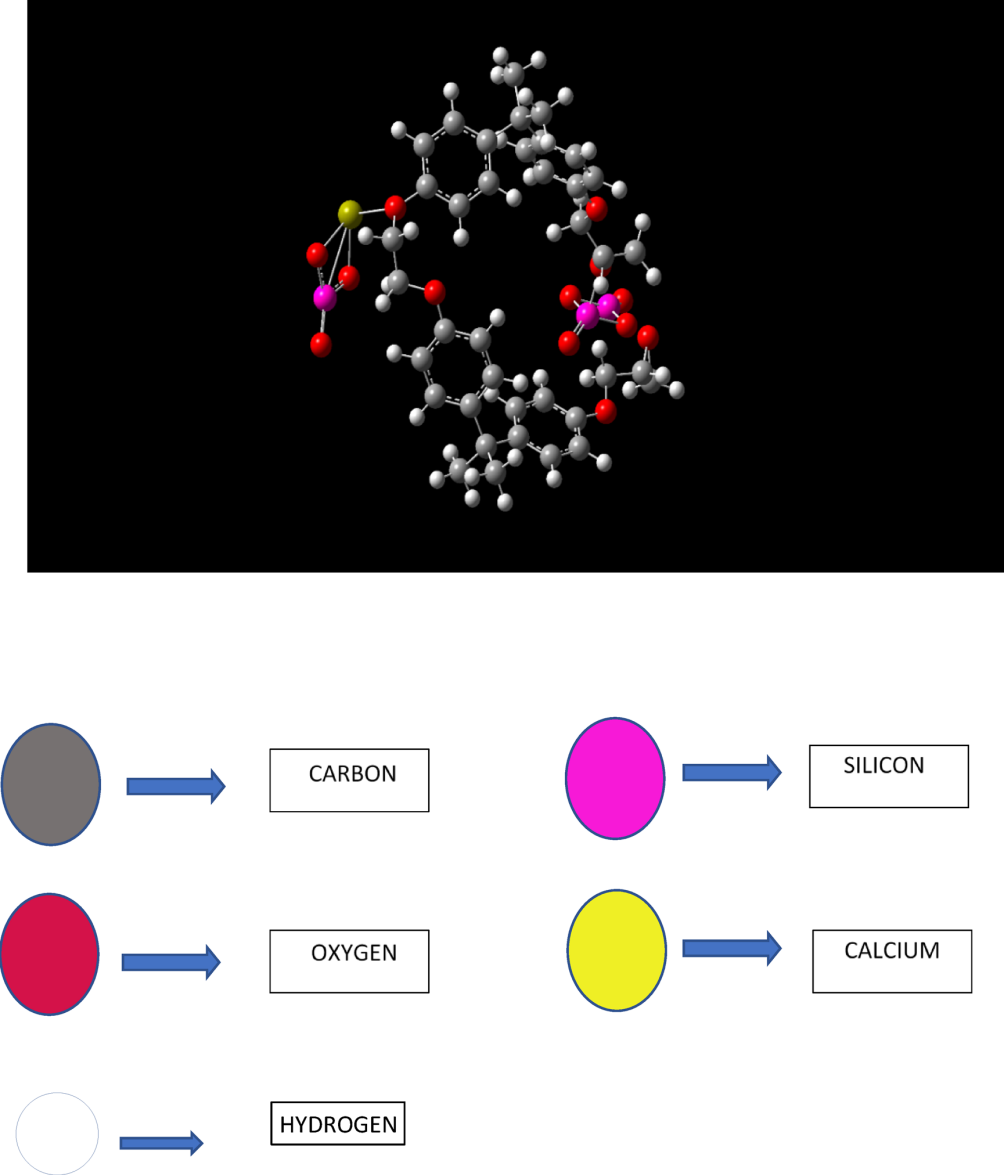
Final structure of the molecule with the filler (wollastonite) attached to the base.

The molecules involved in the actual treatment are taken into the study. Epoxy resin as the main base for the study, wollastonite as the filler material and Silica molecule is taken for E-glass fibre for study. Glass generally is amorphic in nature and modelling for a molecule can only be done if the sample or the molecule under study is crystalline in nature. So, silica being the compound with major composition in the E-glass fibre is taken in for the present study and the interactions had been carried out accordingly. All above the molecules are opted for the study are represented below. Detailed interaction between epoxy molecule, wollastonite and silica had been carried out with the help of density functional theory. The Fig. 5 shows the final representation of the molecule without the binder wollastonite. The geometric optimization when performed minimizes the total energy by adjusting the atomic positions, the final convergence is achieved when the maximum force on any atom is < 0.001 Ry/bohr (i.e. 0.025 eV/A ˙ and the total energy change between the steps is < 1e-4 Ry. These criteria ensure the structure reaches the local minimum, validated by the absence of imaginary frequencies in vibrational analysis, as seen in the FTIR studies at 1017.6 cm-1 indicating stability. The Fig. 6 shows the final representations of the final molecule with the binder wollastonite. Many possible configurations of the final sample containing all the materials such as the epoxy, silica and the binder material wollastonite has been modelled, out of all the configurations of the structures that was calculated post-optimization, with sample A showing 20.67 Debye vs. 12.31 Debye for sample B, suggesting a strong polarity with the filler, the selection process involved modelling multiple configurations, ultimately choosing the one lowest energy and highest dipole moment, reflecting enhanced interactions. Stability is also one of the main deciding factors in picking the right one out, in our process, the one with lower values indicated the highest stability. The stability at the binding sites of the filler material to the original material showed that, Oxygen atoms acted as an intermediate connector between the calcium atoms present inside wollastonite and the silicon atoms present in the glass structure. In the epoxy reactions, oxygen formed an intermediate bond in between the silicon atom and the carbon atom present at the tetra-methyl group. The primary binding site present in the epoxy molecule was at the tetra-methyl carbon, other binding sites such as the carbon atoms at phenyl ring too provided insights about the reactivity, but the structure has a low stability rate for its formation, so that configuration has been ruled out, and the structure shown below has been considered for further study.

The binding energies of the respective molecules at their targeted binding sites were calculated using the equation Eb = Efinal molecule− (Esub-molecule 1 + Esub-molecule 2), where Eb is the binding energy of the final molecule, E_final molecule_ is the energy of the final molecule, and E_sub−molecule 1_ is the energy of the molecule present inside the complex. A Table 3 below depicts bonding energy values of the epoxy with and without the binding material wollastonite.

### Binding energy

When compared theoretically, Bonding value of filler included in final molecule is higher than the bond strength reported without filler. The calculated energies were taken and converted to Ry units. The bond lengths computed here are based on the interaction in between the tetra-methyl carbon atom and the silicon atoms present in the glass structure and the silicon atoms present in the wollastonite structure.

**Table 3:** Binding energy values of the epoxy with and without the binding material wollastonite.

Serial Number	Molecules present in the composite sample	Binding interaction energy (in Ry) (Without filler)	Binding interaction energy (in Ry) (With filler)	Bond length of C-Si bond sample after the filler addition (A^0^)
01	Epoxy (bisphenol A)	510.02	862.30	2.67
02	E - GLASS	404	760.96	2.66
03	Wollastonite	-------	710.57	2.92

. The individual components of the final sample are taken and checked for their binding energies to estimate the stability of the final structure they form without the presence of the filler material. In the other case they are tagged with the filler material individually and their initial binding energies are calculated. The tabulated results above show that the components in the presence of the filler material have increased binding energy thereby making the final sample with the filler material more stable and relatively stronger. One possible reason is epoxy rings are weaker in ring formulation for opening ring mechanism witnessing with reagents. for example- H2O and during this reaction the ring opens and leads to the formation of a glycol with an aliphatic chain in a polymeric compound. During this reaction the epoxide at the end of the chains is attacked by a nucleophile which results in the generation of an alkoxide and in its next step forms a glycol product based on the reagent addition^21^.

**Table 4:** C-Si length of bind & corresponding binding energies post interaction with target molecule.

Serial Number	Samples at test	E_DFT_ [ RB3LYP] in au	Dipole Moment in Debye	Bond length of C-Si bond sample (A^0^)
01	Sample A (Bisphenol-A + SiO2 + CaSiO3)	-3996.590	20.67	3.35005
02	Sample B (Bisphenol-A + SiO2)	-2338.178	12.31	2.82642

The final sample structures have been subjected to geometric optimization and the final energies which can be linked to their stabilities are given above in the Table 4. The negative sign indicate that the reaction performed is exothermic in nature. The sample with the filler material bonding has a better performance rate as supported by the experimental results. In the present simulation the sample with the filler material has better stability and better dipole moment. The addition of wollastonite as a filler introduces Ca^+^ ions that interact with the O_2_ atoms in the C-O-Si linkages. As a result, the effective distance between the carbon and silicon atoms increases that leads to the longer bond lengths in the composite with filler compared to without. For instance, the current studies show that the C-Si bond length increasing from 2.82$\:\dot{A}$ to 3.35 $\:\dot{A}$ with filler addition, The current change can be attributed to several factors including the electronic effects where in Ca with an electronegativity of 1.0, forms an ionic or coordinate bonds with oxygen, potentially withdrawing electron density from the C-O-Si bridge which alters the bond order or strength, lengthening of the effective C-Si distance. Also, the presence of Ca + ions might induce steric hindrance or strain, changing the geometry of the C-O-Si linkage, which could stretch. NMR analysis of the final sample is done on the to calculate the chemical shifts at various binding sites. Gaussian 09 does a shielding tensor calculation to estimate the NMR spectrum of the sample. It uses Gauge-Independent Atomic Orbital Method (GIAO) to compute the isotropic shielding constant for each nucleus. This reflects how the local electron density shields the nucleus from the effects of the external magnetic field. The chemical conversion () is calculated relative to a reference compound via:

$$\:\delta\:\:=\:{\sigma\:}_{ref}\:-\:\sigma\:$$

Where$\:\:{\sigma\:}_{ref}$ is the shielding constant of the reference taken (here the values for O used in the above structure are taken from the oxygen in H_2_O structure. Gaussian 09 gives us the shielding constants that are converted to chemical shifts (in ppm) for plotting the NMR spectrum, their respective graphs are shown below.

**Fig. 7:**
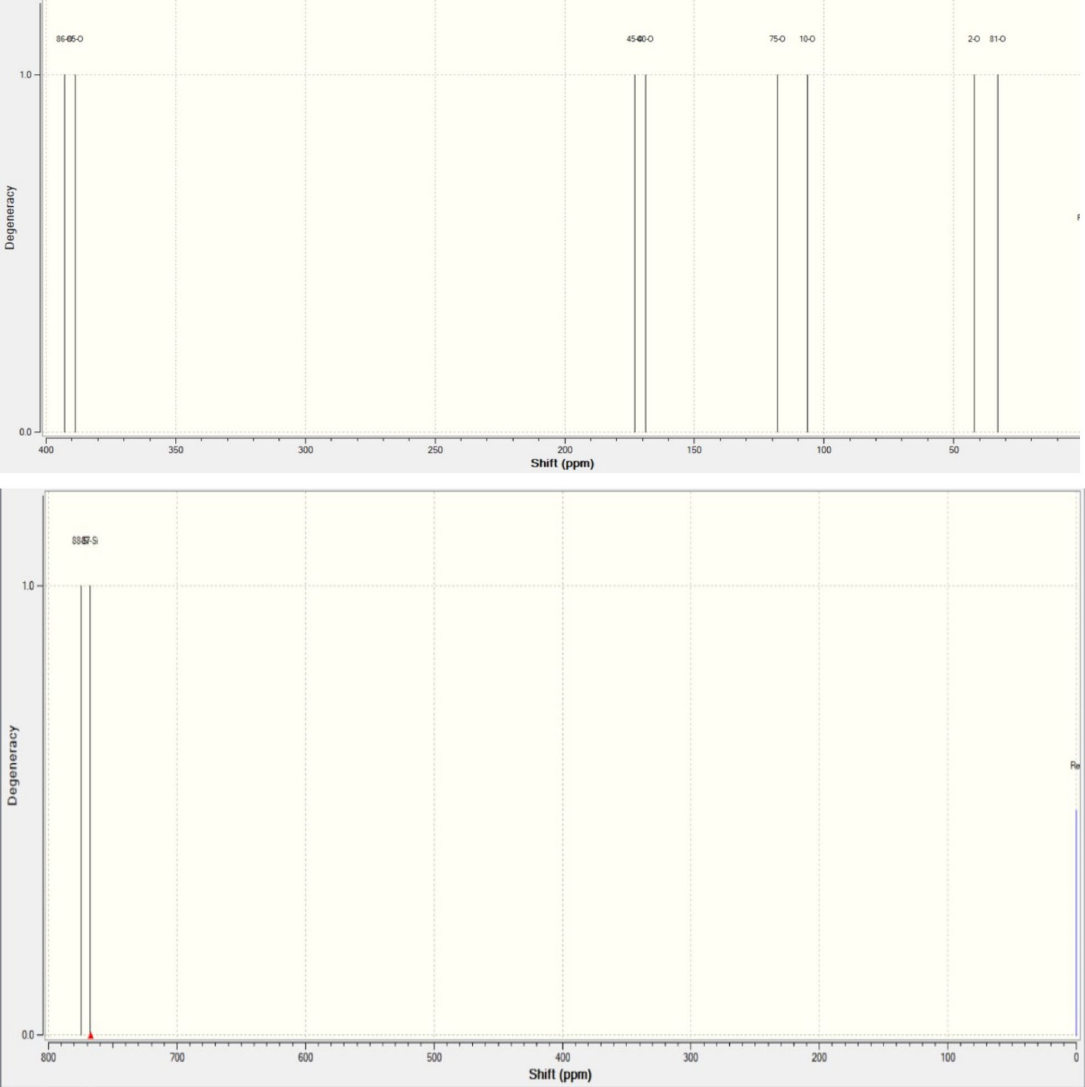
Computed NMR shifts of oxygen and silicon atoms present in the sample.

These sites being unknown gives us a peak whenever we observe a chemical shift due to the interaction in between 2 different atoms or same atoms with multiple conditions such as steric hindrance, dipole moment, etc. The computed sample in the Fig. 7 of the composite showed a highest peak at degeneracy 1.0, at multiple chemical shifts for oxygen atoms. The reference function used here is the H_2_O B3LYP/6-311 + G (2d, p) under SCF-GIAO method, the reference shielding values used for oxygen was 320. The oxygen atoms 86-O, 85-O,81-O,75-O, 45-O,40-O,10-O, and 2-O showed a chemical shift of 392.724 ppm, 388.673 ppm, 33.011 ppm, 118.067 ppm, 173.018 ppm, 168.717 ppm, 106.471 ppm, 41.9505 ppm all at the same degeneracy of 1.0. the difference level was 0.5 which was acceptable when compared to the reference level. Multiple peaks for the O_2_ spectrum indicate a diverse environment, like the high shifts ( e.g., 392.724 ppm) may indicate that the O_2_ atoms with lower electron density, possibly coordinated with calcium, leading to a downward shift due to electron withdrawal. The silicon atoms on the other hand produced the same difference in degeneracy with the reference level. The 88-Si, 87-Si has showed a chemical shift 774.791 ppm and 767.706 ppm the reference functions are taken as previous cases. The reference shielding value used for Si atoms under the same scheme was 327.3890. For silicon, shifts at 774.791 ppm and 767.706 ppm indicate different environments, likely SiO2 and CaSiO3, with calcium altering silicon’s electronic structure. Carbon shifts (130–160 ppm) for aromatic or carbonyl carbons suggest interface interactions, with 37-C and 67-C possibly near C-O-Si linkages, experiencing electron density changes from calcium coordination, reflecting new bond formations. These likely reflect different environments (e.g., SiO2 vs. CaSiO3), with the filler’s calcium altering the electronic structure.

The NMR shifts correlate explicitly with molecular interactions, particularly calcium ion effects. For oxygen, higher ppm shifts (e.g., 392.724 ppm) suggest coordination to calcium, reducing electron density and causing downfield shifts, as seen in oxometalates where electropositive ions lead to up field shifts for more bound oxygen which is also observed from the previous study^25^. However, in this context, coordination may increase δ due to deshielding.

**Fig. 8:**
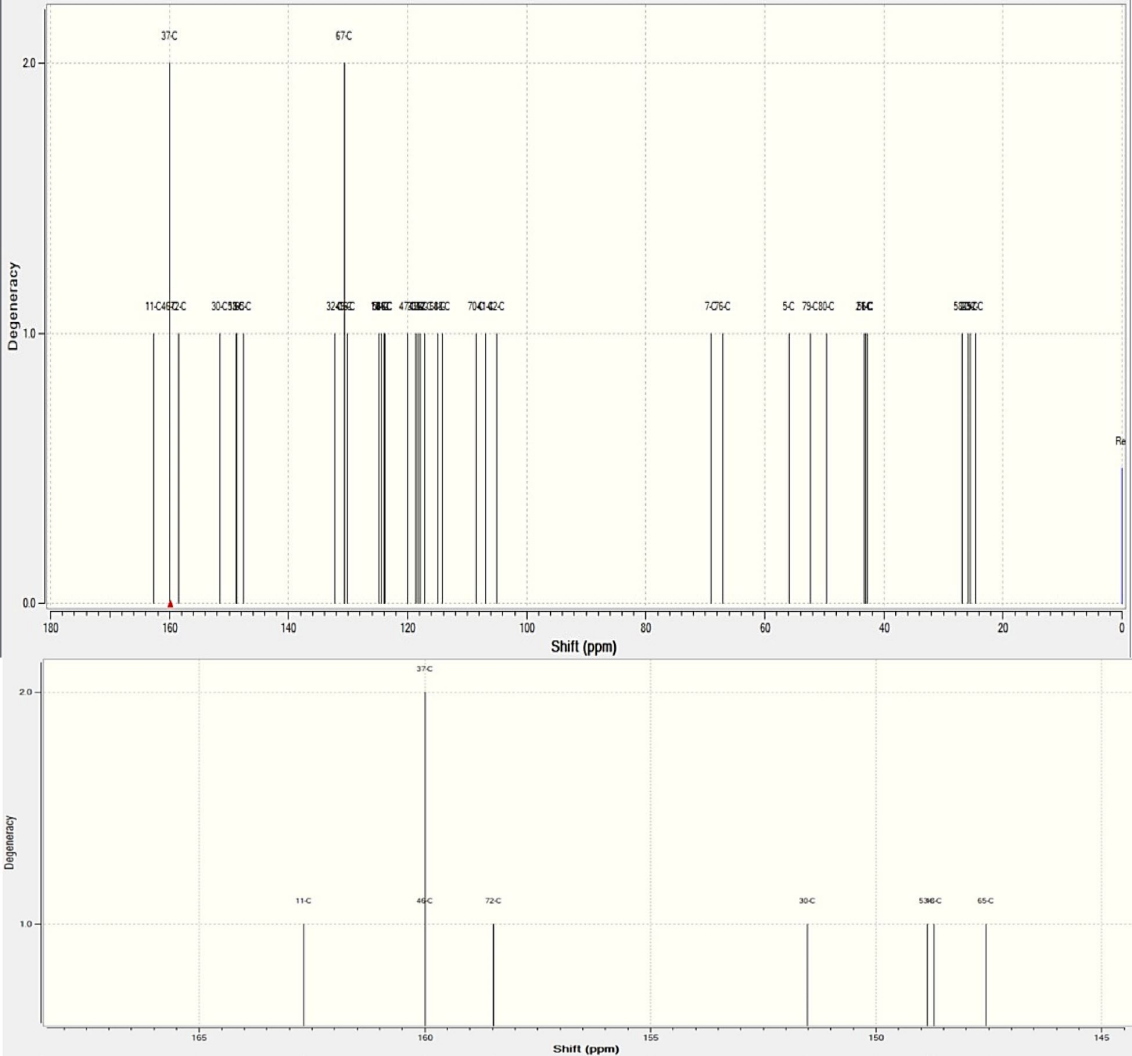
(**a**) The computed NMR chemical shifts for Carbon atoms, (**b**) The NMR chemical shifts of the region (magnified).

For Carbon as shown above in the Fig. 8 the reference shielding value was taken at 182.4656. The chemical shift in the carbon atoms however, revealed a point about extra stretching at the 37-C and 67-C due to the addition of the Ca atoms and its variation in the bond length, the degeneracy levels reached 2.0 for these atoms and their shifts were considerably higher when compared to the carbon atoms at other positions. The shifts of these atoms are 160.486 ppm and 130.619 ppm. The analysis revealed a point that the carbon atoms with the shifts similar in their region around those with higher degeneracy levels are relative in frequency in the structure. The atoms 11-C, 46-C, 72-C have a closely relative chemical shifts 162.691 ppm, 159.996 ppm, 158.454 ppm with the 37-C, this can be observed by the Fig. 8(b). Ingrid et al. and their team in their works has evaluated the shifts over NMR for various antioxidants in DMSO with the help of B3LYP calculations and identified that the ascending number in chemical shifts might be corelated with ascending reactivity at the site^22^. These shifts, typical for sp-2 carbons (aromatic or carbonyl), indicate carbons near the interface, possibly involved in C-O-Si linkages, affected by calcium interactions. Changes in these electronic shifts of these atoms are because of interactions with the filler are indicated by the observed changes in chemical shifts following filler addition. Higher chemical shifts for silicon and carbon atoms in particular indicate a more de-shielded state, which could be the consequence of new bonding connections formed by the filler or electron removal by calcium ions. The broad range of chemical shifts for oxygen atoms (41.9505 ppm to 392.724 ppm) is indicative of a variety of settings, including silicate bridges, carbonyl groups, and ether connections. The observed NMR chemical shifts can be directly correlated with specific molecular interactions introduced by the wollastonite filler. For oxygen atoms, shifts corresponding to Ca-O bonds indicate the formation of new interactions between calcium ions and oxygen atoms in the composite, such as those in the C-O-Si bridges or other oxygen-containing groups. These interactions alter the electronic environment around the oxygen atoms, leading to changes in their chemical shifts. For carbon atoms, changes in chemical shifts suggest modifications in their bonding or proximity to the filler, possibly through electron withdrawal or new bond formations facilitated by the filler. Similarly, for silicon atoms, shifts in their resonances reflect alterations in the silicate structure or their coordination environment due to the presence of calcium ions.

**Fig. 9:**
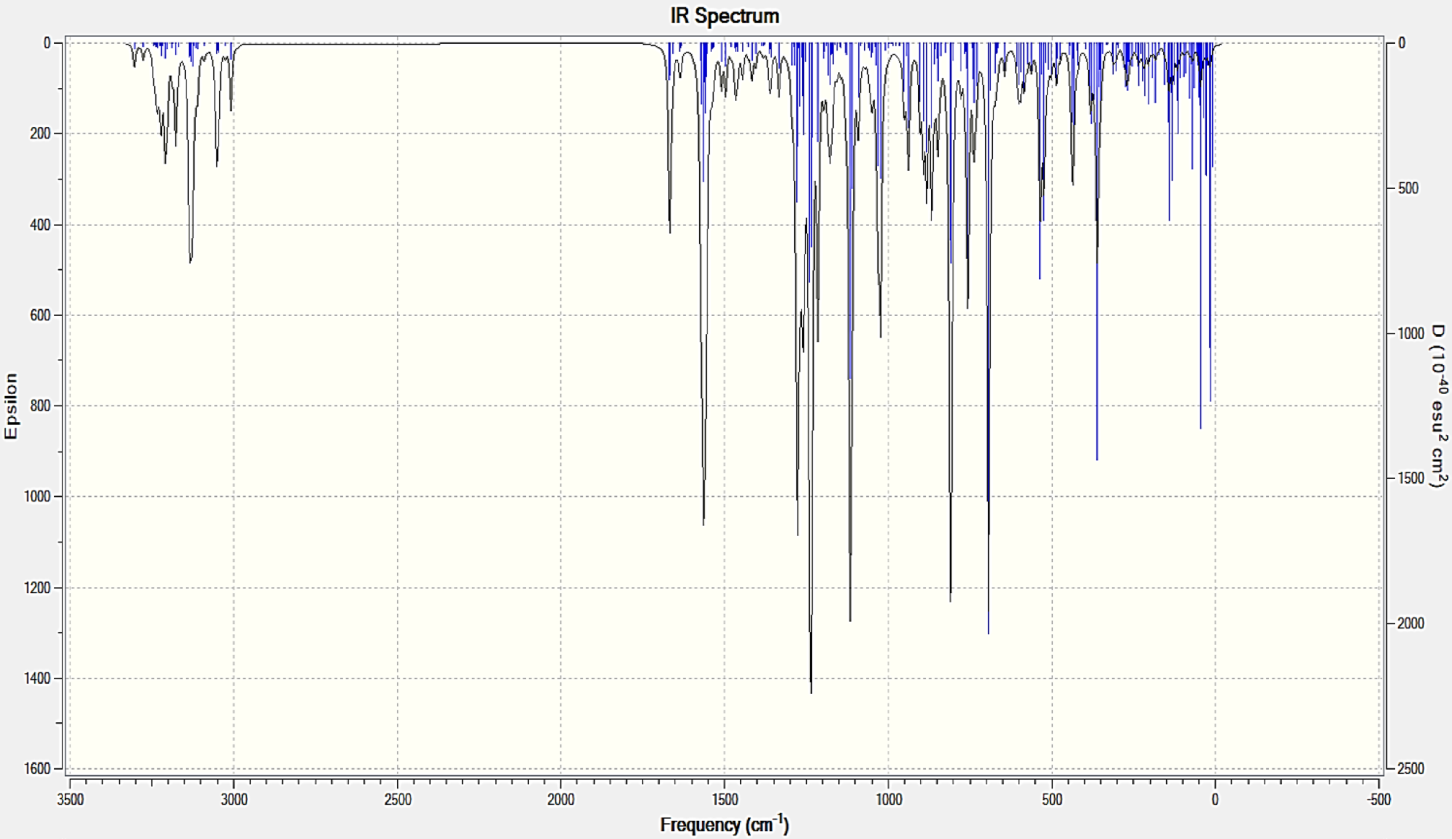
FTIR spectrum of the composite (computed).

**Fig. 10:**
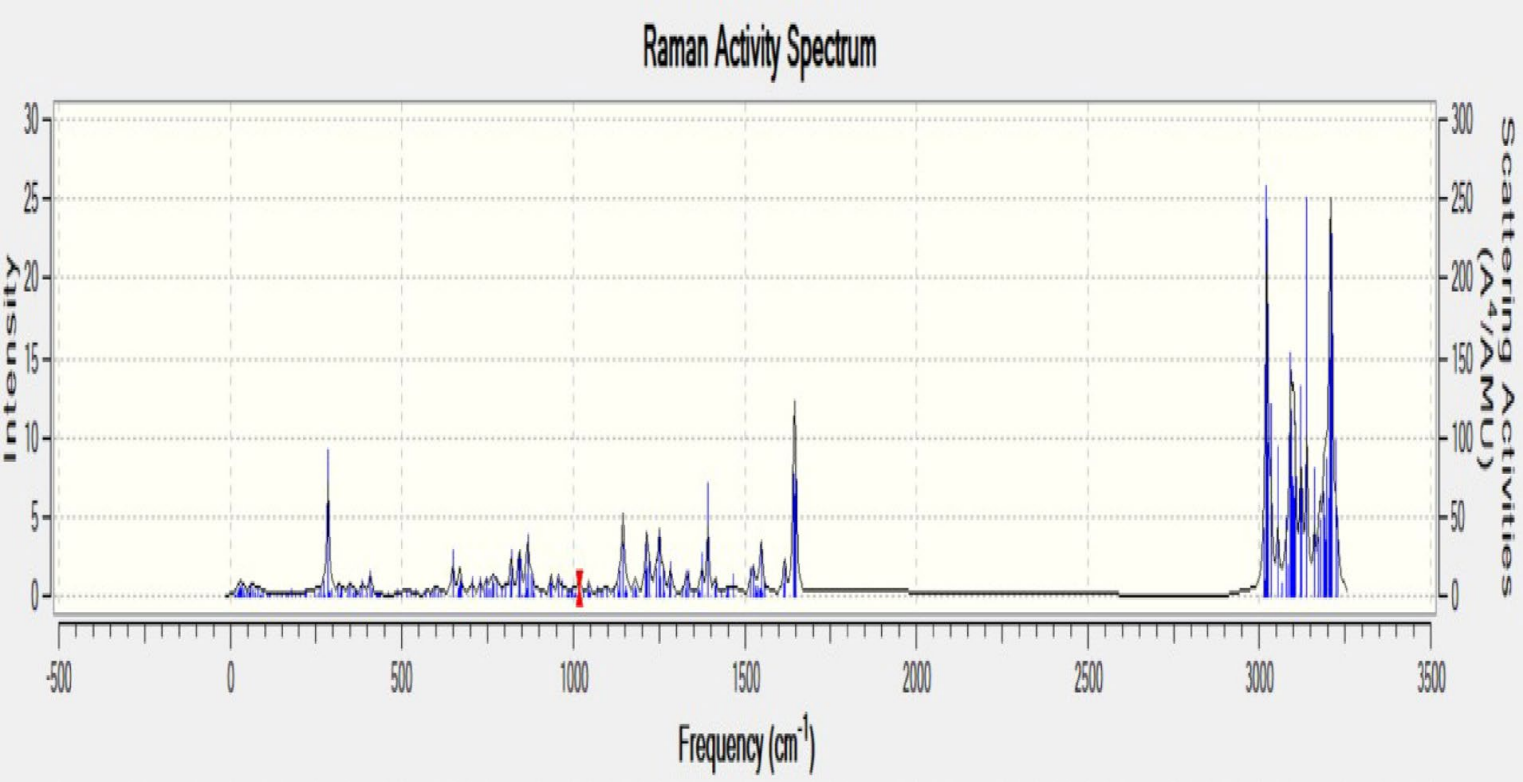
The Raman spectrum graph (computed) for composite with the peak deflections.

For the present analysis, sample examined at 285 modes & Spectrum plot of IR was drawn accordingly. Concluding with peak value is reported for 1017.6 cm^− 1^ frequency at the 159th mode. One of the outcomes of this analysis is to inspect for additional presumable range of frequencies. These range demonstrates the peak locality with higher value maintain the energy contour in the stored energy form. However, existence imaginary frequency range denotes profound negative eigen value within the equation while performing the computational analysis. Results in imaginary frequency at any of the mode, however present interpretation does not indicate any involvement of imaginary frequencies. When other configurations of the final sample were tested, a few of the structures gave imaginary frequencies which doubted their possible existence outside the theory. Most of those structures were filtered while choosing the final structure for the sample. The present configuration of the sample has been created in the presence of vacuum. The actual FTIR results shown in Fig. 9 match with the computed ones with slight changes in its absorption bands. Now, this change is due to the presence of additional bands occurred due to the nucleophilic addition which resulted in a possible epoxide ring opening reaction. But this occurs only in the presence of a reagent and since there are no reagents added in the present reaction, the only possible explanation for the presence of additional bands in the original FTIR spectrum is due to the formation of additional by-products such as water and an alcohol in a disproportionate amount throughout the sample which resulted in the epoxide ring opening at some parts leading to the formation of a straight chain glycols. The major difference between the samples in the presence of the filler material and in the absence of a filler material is their binding energy which ultimately can help us determine their stability and reactivity. In general, the methyl group vibrations are downshifted in the simulation to counter the electronic effects from the carbonyl groups formed in the process of ring opening. But if the error margin is below 10%^23,26–30^. The Raman graph as shown in Fig. 10 below agrees with the above proposed frequency analysis, Raman activity results due to the change of polarizability for a molecule, and the Raman spectrum analysis at the frequency ranges 3000 cm^− 1^ to 3200 cm^− 1^ showed much more deflections in the form of ranging peaks, the highest peak produced at 3020 cm^− 1^ with the highest scattering activity of 257.558 (A^4^/AMU), at the frequency 3020.96 cm^− 1^. The band has narrowed from 1600 till 3000 frequency range, can depict us the low vibration of the C-Si.

**Fig. 11:**
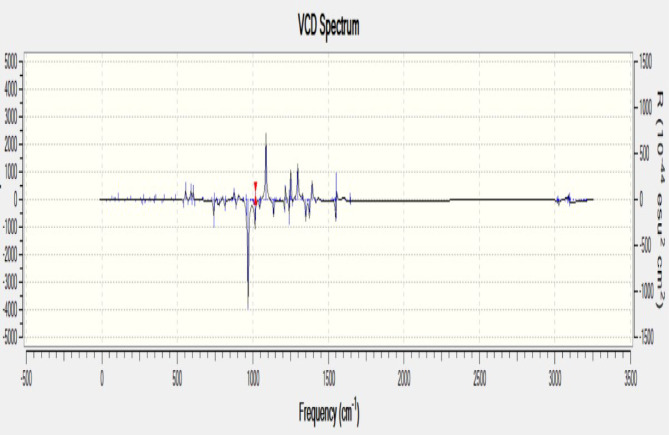
Vibrational circular dichroism (VCD) spectra of composite.

Computed Vibrational circular Dichroism spectrum (VCD) has agreed with the statement above, Fig. 11 shows us info about some of the important features of the sample, such as the dipole moment and the best stable configuration among others. In general, this analysis is used in the determination of measurements of a particular configuration such as the dipole moment, it is also very helpful to calculate the absolute configuration of a molecule, the enantiomeric excess of chiral molecules, and the energy of the structure based on the function used and the basis set used. The peak of the graph is observed at 1086.58 cm ^− 1^ frequency and at *R* = 638.635 esu^2^ cm^2^. The increasing trend of R is the increasing enantiomeric purity of R configuration of the sample, in the same way the decreasing trend of R is equal to the increasing trend of S configuration on the y-axis.

### Combined Spectro-scopic analysis

The combined Spectro-scopic analysis (FTIR, Raman, and VCD) clearly represents wollastonite, an active filler which significantly enhances the binding strength within the composite matrix. In the FTIR spectrum, more intense with distinct bands below 1200 cm^− 1^ correspond to Si–O–Si and Ca–O stretching modes which confirms the presence of the wollastonite framework^31^. Shifts and intensity variations in the hydroxyl and carbonyl regions suggest strong interactions between the filler and functional groups in the matrix, likely through hydrogen bonding or coordination^32^. Complementary Raman spectra display sharp and intense peaks in the 900–1100 cm^− 1^ region associated with symmetric Si–O vibrations, implying with enhanced crystallinity and molecular alignment due to filler addition. Additional bands in the 2900–3100 cm^− 1^ region point to the presence of organic C–H groups, whose altered intensity reflects changes in molecular environment due to filler and matrix interactions. The VCD spectrum further supports this by showing well-resolved signals in the same fingerprint region, indicating that wollastonite induces conformational changes or asymmetric organization in the surrounding organic matrix. The cumulative effect of these structural features is consistent with enhanced mechanical performance. For instance, composites reinforced with E-glass fiber and 1–5% wollastonite showed improved tensile and flexural strength, attributed to better interfacial adhesion and dispersion of the filler^33^. Furthermore, in polypropylene-based systems, wollastonite was found to increase the onset of thermal degradation and promote crystallinity, likely due to its nucleating ability and strong polymer-filler interaction^34^.

##  Conclusion

Present study demonstrates the preparation of wollastonite filled GFRP composites with varied filler percentage. Study investigates the binding nature of epoxy resin with mineral with glass fiber, using DFT simulations and spectral analyses. Present work concludes in understanding how wollastonite improves interfacial bonding by facilitating stronger interactions between the epoxy matrix and the E-glass fiber by enhancing composite stability. Epoxy resin molecules are generally non-polar in nature, and in general the binding forces that essentially form in between non-polar molecules are weakest in nature making them viable to break under the application of minimal external force. So, to avoid this conflict in the real time experiment, wollastonite is added as a filler material to decrease the intermolecular repulsion and increase the bonding capacity inside the sample containing E-glass fibre and epoxy resin. Spectral simulation studies such NMR suggested that the major chemical shifts in the 37 and 67 carbon atom suggested a new interaction in between the calcium atoms in the wollastonite and the carbon atoms of the epoxy molecule and the silicon atoms from the glass fibre structure, by calculation of the binding interaction energy there was an increase in the interaction energy by 352.3 Ry in the epoxy resin molecule individually and the increase in the binding interaction energy in the e-glass fibre alone amounted to 356.955 Ry. Even the Raman spectrum activity depicted the same, the minor change in the polarizability of the molecule resulted in the new bond formation with the filler material and this led to an increase in the frequency by 200 cm^− 1^, and as the system reached to a temporary equilibrium after the increase in the frequency due bond formation, the system flattened for a period of frequency from 1600 to 3000 frequency and rose again due to the new bond formation with another filler molecule. These results witness wollastonite as the filler material increased bonding strength in the individual components thereby increasing the strength of the material. Additionally, combined spectroscopic analysis (FTIR, Raman, and VCD) provided further support for these interactions. Raman spectra showed frequency shifts (~ 200 cm^− 1^) linked to new bond formation, while VCD and FTIR indicated molecular reorganization and enhanced binding with the filler, confirming improved cohesion at the molecular level. The molecular insights of the study correspond to wollastonite enable, fine-tuning of composite properties by enhancing interfacial bonding between epoxy and E-glass fiber. This results in improved molecular cohesion, stability, and interaction. Such enhancements pave the way for smart, durable, and high-performance material applications.

## Data Availability

The datasets during and/or analyzed during the current study are available from the corresponding author upon reasonable request.
